# *In vitro* anti-Herpes simplex virus activity of crude extract of the roots of *Nauclea latifolia* Smith (Rubiaceae)

**DOI:** 10.1186/1472-6882-13-266

**Published:** 2013-10-16

**Authors:** Manuela Donalisio, Huguette Magnifouet Nana, Rosalie Annie Ngono Ngane, Donatien Gatsing, Alembert Tiabou Tchinda, Roberta Rovito, Valeria Cagno, Cecilia Cagliero, Fabrice Fekam Boyom, Patrizia Rubiolo, Carlo Bicchi, David Lembo

**Affiliations:** 1 Laboratory of Molecular Virology, Department of Clinical and Biological Sciences, University of Turin, PO Box 10043, Torino, Italy; 2 Laboratory of Microbiology and Antimicrobial Substances, University of Dschang, PO Box 67, Dschang, Cameroon; 3 Laboratory of Biochemistry, University of Douala, PO Box 24157, Douala, Cameroon; 4 Laboratory of Phytochemistry, Institute of Medical Research and Medicinal Plants Studies, PO Box 6163, Yaoundé, Cameroon; 5 Laboratory of Phytoanalysis, Department of Drug Science and Technology, University of Turin, PO Box 10125, Torino, Italy; 6 Laboratory of Phytobiochemistry and medicinal plants study, Department of Biochemistry, University of Yaoundé, PO Box 812, Yaoundé, Cameroon; 7 Department of Clinical and Biological Sciences, University of Turin, S. Luigi Gonzaga Hospital, Regione Gonzole, 10, 10043 Orbassano, Turin, Italy

**Keywords:** *N. latifolia* roots, CH_2_Cl_2_/MeOH extract, Phytochemistry, Antiviral activity, HSV-2

## Abstract

**Background:**

*Nauclea latifolia* Smith*,* a shrub belonging to the family Rubiaceae is a very popular medicinal plant in Cameroon and neighboring countries where it is used to treat jaundice, yellow fever, rheumatism, abdominal pains, hepatitis, diarrhea, dysentery, hypertension, as well as diabetes. The ethno-medicinal use against yellow fever, jaundice and diarrhea prompted us to investigate on the antiviral activity of the root bark of *N. latifolia*. In this study, HSV-2 was chosen as a viral model because of its strong impact on HIV transmission and acquisition.

**Methods:**

The crude extract under study was prepared by maceration of air-dried and powdered roots barks of *N. latifolia* in CH_2_Cl_2_/MeOH (50:50) mixture for 48 hours, then it was subjected to filtration and evaporation under vacuum. A phytochemical analysis of the crude extract was performed by High Performance Liquid Chromatography coupled with a photodiode array and mass spectrometry (HPLC-PDA-ESI-qMS). The anti-HSV-2 activity was assayed *in vitro* by plaque reduction and virus yield assays and the major mechanism of action was investigated by virucidal and time of addition assays. Data values were compared using the Extra sum of squares F test of program GraphPad PRISM 4.

**Results:**

The main components detected in the extract belong to the class of indole alkaloids characteristic of *Nauclea* genus. Strictosamide, vincosamide and pumiloside were tentatively identified together with quinovic acid glycoside. *N. latifolia* crude extract inhibited both acyclovir sensitive and acyclovir resistant HSV-2 strains, with IC_50_ values of 5.38 μg/ml for the former and 7.17 μg/ml for the latter. The extract was found to be most active when added post-infection, with IC_50_ of 3.63 μg/ml.

**Conclusion:**

The results of this work partly justify the empirical use of *N. latifolia* in traditional medicine for the treatment of viral diseases. This extract could be a promising rough material for the development of a new and more effective modern anti-HSV-2 medication also active against acyclovir-resistant HSV-2 strains.

## Background

Genital herpes is a widespread sexually transmitted infection, caused by the herpes simplex virus (HSV), most commonly of type 2 (HSV-2) [1]. After the primary infection, HSV-2 is transported retrogradely to the lumbosacral sensory ganglia, where it establishes a lifelong latent infection that can be reactivated by stress, hormonal changes and UV light. After reactivation, HSV-2 can be transported to the primary site of infection causing either asymptomatic episodes, which facilitate its spread in the population, or recurrent ulcerations to the genital mucosa. These lesions are often very painful and can lead to substantial psychological morbidity [2]. The virus can also be passed from mother to child during birth with the risk of very serious neonatal infections [3]. It has been estimated that the global prevalence in people aged 15–49 years who were living with HSV-2 worldwide in 2003 was 536 million whereas the estimated number of new HSV-2 infections among 15–49 year olds worldwide in 2003 was 23.6 million [4]. The incidence of HSV recurrence is increased in people with an impaired immune system, such as HIV-seropositive individuals and in transplant recipients [5,6]. On the other hand, genital herpes may increase the risk of HIV acquisition by disrupting epithelial cells, with induction of local inflammation and production of cytokines and chemokines that activate and recruit CD4^+^ HIV target cells [7]. In a systematic review including a meta-analysis of longitudinal studies, prevalent HSV-2 infection was associated with a three-fold increased risk of HIV acquisition in both men and women, suggesting that, in areas of high HSV-2 prevalence, a high proportion of HIV infection is attributable to HSV-2 [8]. Therefore, strategies that can prevent or treat HSV infections are expected to reduce rates of sexual HIV transmission [9]. Currently no vaccine is available. The standard therapy for management of HSV infections is based on nucleoside analogues that target the viral DNA polymerase. These include acyclovir, penciclovir and their derivates, valacyclovir, and famciclovir [10,11]. These antiviral drugs can be efficacious to treat clinical signs and symptoms of first and recurrent episodes, but their widespread use and the long term prophylactic therapy may be associated with relative high toxicity and emergence of drug-resistant virus strains especially in immunocompromised patients [12]. For these reasons, there is a great demand for the development of new antiviral drugs with novel mode of action. In this context, natural products from medicinal plant extracts are very important source of anti-HSV agents and several extracts and pure compounds from herbal medicines have been reported to exert an anti-HSV activity [13].

African plants constitute a rich and still underexplored source of natural products of potential medical interest [14]. *Nauclea latifolia* Smith (syn: *Sarcocephalus latifolius* (Sm.) Gruce) is a straggling shrub or small tree of about 4 m high abundantly spread in all inter-tropical Africa. In Cameroon, the roots are used to treat jaundice, yellow fever, rheumatism, abdominal pains and hepatitis and the bark to treat jaundice and loss of appetite [15]. In Nigeria, the stem bark and roots of the plant are used against fever, jaundice, malaria, diarrhea, dysentery, hypertension and diabetes [16]. Pharmacological studies of *N. latifolia* have shown antibacterial [16], antidiabetic [17] and antiplasmodial [18] activities. Previous phytochemical studies on *N. latifolia* have yielded a great number of indole alkaloids, triterpenes, steroids and saponins [19-21]. The traditional use against yellow fever, jaundice and diarrhea prompted us to investigate on the antiviral activity of the root bark of *N. latifolia*. The present study was undertaken to test the crude extract of *N. latifolia* for its antiviral activity against acyclovir-sensitive and resistant strains of HSV-2 and to investigate its probable mode of action.

## Methods

### Plant material

The root barks of *N. latifolia* were collected in January 2011 at Makenéné (Centre region, Cameroon) and identified at the National herbarium of Yaoundé (Cameroon) where a voucher specimen (No 58281/HNC) was deposited.

#### Preparation of the crude extract

The air dried and powdered root barks (1 Kg) of *N. latifolia* were macerated with CH_2_Cl_2_-MeOH mixture (6 L) for 48 hrs. Filtration and removal of solvent under vacuum using an evaporator gave a brown extract (30 g) which was subsequently subjected to phytochemical and biological assays.

### HPLC-PDA-MS analysis

*N. latifolia* crude extract was analyzed by a Shimadzu LC-MS 2010EV system equipped with a photodiode detector SPD-M20A (Shimadzu, Dusseldorf Germany) in series to a single quadrupole MS system provided with orthogonal atmospheric pressure chemical ionization (APCI) and electrospray ionization (ESI) sources. An Ascentis Phenyl column (250 × 4.6 mm i.d., 5.0 μm), (Supelco, Bellefonte, PA) was used. Analysis conditions were: temperature: 40°C; mobile phase: eluent A: water; eluent B: acetonitrile; mobile phase gradient was as follows: 20-50% B in 37.5 min, 50-90% B in 16.67 min, and 90% B for 3 min. Injection volume: 10 μl, flow rate: 0.4 ml/min. UV spectra were acquired in the 210–450 nm wavelength range and the resulting chromatograms were integrated at different wavelengths in function of the UV absorption maxima of each component. MS operative conditions: ESI temperature: 200°C; nebulizer gas flow rate: 1.5 ml/min; curve desolvation line (CDL) temperature: 250°C. Mass spectra were acquired both in positive and in negative full-scan mode in the range of 150–900 m/z, with a scan range of 1000 u/s.

Identification of the components was based on their UV spectra, and mass spectral information compared to those reported in literature with which are in full agreement.

### Cell lines and viruses

African green monkey kidney cells (Vero) (ATCC CCL-81) were cultured in Eagle’s minimal essential medium (MEM) (Gibco/BRL, Gaithersburg, MD) supplemented with heat-inactivated 10% foetal calf serum (FCS) (Gibco/BRL) and 1% antibiotic-antimycotic solution (Zell Shield, Minerva Biolabs GmbH, Berlin, Germany), at 37°C in an atmosphere of 5% of CO_2_.

A clinical isolate of HSV-2, sensitive to acyclovir was kindly provided by Prof. M. Pistello, University of Pisa, Italy. A HSV-2 strain with phenotypic resistance to acyclovir was generated by serial passage in the presence of increasing concentrations of acyclovir as previously described by Field [22]. The resistant virus was then plaque-purified, and the antiviral susceptibility was tested by a plaque reduction assay showing an inhibitory concentration producing 50% reduction in plaque formation (IC_50_) of 319 μM. Viral strains were propagated and titrated by plaque assay on Vero cells, as previously described [23].

### Reagents

Heparin (13.6 kDa) was obtained from Laboratori Derivati Organici Spa (Milan, Italy). Acyclovir (Sigma-Aldrich).

### Cell viability assay

Vero cells were seeded into 96-well plates at a density of 10^4^ cells/well, and incubated at 37°C in a 5% CO_2_ atmosphere for 24 h. Increasing concentrations of the test extract were added to the cells, with a replicate number of three wells per concentration. After a two day incubation period, cell viability was measured by the MTS [3-(4,5-dimethylthiazol-2-yl)-5-(3-carboxymethoxyphenyl)-2-(4-sulfophenyl)-2H-tetrazolium] assay by the CellTiter 96 Proliferation Assay Kit (Promega, Madison, WI,USA) according to the manufacturer’s instructions. Absorbances were measured using a Microplate Reader (Model 680, BIORAD) at 490 nm. The resulting cell viability was calculated as described previously [24]. The 50% cytotoxic concentrations (CC_50_) and 95% confidence intervals (CI) were determined using GraphPad PRISM software (Graph-Pad Software, San Diego, CA).

### HSV antiviral assays

Inhibition of HSV replication was evaluated with plaque reduction assay and virus yield reduction assay. Vero cells were seeded in 24-well plates at a density of 10 × 10^4^ cells. The plaque reduction assay was performed infecting cell monolayers with HSV-2 or HSV-2 mutant with phenotypic resistance to acyclovir at a multiplicity of infection (MOI) of 0.001 pfu/cell in presence of serial dilutions of the test extract for 2 hours at 37°C. The inocula were subsequently removed from the wells, and the cells were washed with medium twice and overlaid with a medium containing 1.2% methylcellulose (Sigma) and the plant extract. Treatment of control samples with equal volumes of DMSO was performed in order to rule out the possibility of any cytotoxic effect ascribable to the solvent. After incubation for 24 hours at 37°C in 5% CO_2_, the supernatant was removed, and the cells were fixed and stained with 0.1% crystal violet in 20% ethanol and viral plaques were counted. The IC_50_ values, defined as the inhibitory concentration producing 50% reduction in plaque formation, were calculated by using the program GraphPad PRISM 4 (GraphPad Software, San Diego, California, U.S.A.) to fit a variable slope-sigmoidal dose–response curve. A selectivity index (SI) was calculated by dividing the CC_50_ by the IC_50_ values. For the virus yield reduction assay, cells were infected in duplicate with HSV-2 at a MOI of 0.01 pfu/cell in presence of serial dilutions of extract. Following virus adsorption (2 hours at 37°C), the virus inoculum was removed and cultures were exposed to the extract and incubated until control cultures displayed extensive cytopathology. Supernatants were pooled as appropriate 48 hours after infection and cell-free virus infectivity titers were determined in duplicate by the plaque assay in Vero cell monolayers. The end-points of the assay were the inhibitory concentration of extract which reduced virus yield by 50% (IC_50_) in comparison to the untreated virus control.

### Virucidal assay

The direct effect of *N. latifolia* extract on infection of HSV-2 was evaluated by incubating 33 μg/ml of extract with HSV-2 (10^5^ pfu) at either 4 or 37°C for various lengths of time as previously described [25,26]. After incubation, the residual virus infectivity was determined by titration on Vero cells at high dilutions, at which the extract was not active.

### Pre-treatment assay

The pre-treatment was performed treating Vero cells in 24-well plate with different concentrations of extract for two hours at 37°C prior virus infection. After washing, cells were infected with HSV-2 at a MOI of 0.001 pfu/cell for two hours. The infected cells were washed and treated as described above for plaque reduction assay.

### Attachment assay

The attachment assay was performed as previously described [25,26] by mixing *N. latifolia* extract or heparin with HSV-2 (MOI of 0.004 pfu/cell ) at 4°C. The mixtures were then added to cooled Vero cells in 96-well plates and incubated for 2 h at 4°C to allow attachment but not entry. After three washes with cold MEM to remove unbound virus, cells were overlaid with medium containing 1.2% methylcellulose and shifted to 37°C. After 24 hours of incubation, cells were stained and viral plaques were counted. As control, cells were infected in presence of equal volumes of DMSO setting 100% of infection; instead, to confirm viral attachment but not viral entry at 4°C, after incubation cells were treated for two minutes with cold acidic glycine (100 mM glycine, 150 mM NaCl, pH 3) to inactivate attached virus, resulting in 100% inhibition of infection.

### Entry assay

For entry assay, HSV-2 at a MOI of 0.004 pfu/cell was adsorbed for 2 hours at 4°C on prechilled confluent Vero cells. Cells were then washed with cold MEM three times to remove unbound virus, treated with different concentrations of extract, and incubated for three hours at 37°C. Unpenetrated viruses were inactivated with acidic glycine for 2 minutes at room temperature, as previously described [25]. Cells were then washed with warm medium three times and treated as described above for plaque reduction assay.

### Post-treatment assay

For the post-treatment assays Vero cells monolayers were infected with HSV-2 for two hours at 37°C, followed by two gentle washes to remove unbound viruses. Increasing concentrations of extract were then added to cultures. The IC50 values were measured by plaque reduction assay and virus yield reduction assay, as described previously.

### Statistics

IC_50_ values were compared using the Extra sum of squares F test of program GraphPad PRISM 4 (GraphPad Software, San Diego, California, U.S.A.). Values of p < 0.05 were considered indicative of statistical differences. Results represent the mean of three independent experiments ± standard deviations.

## Results

### Phytochemical analysis

*N. latifolia* belongs to the Rubiaceae family which is characterized by the presence of different classes of secondary metabolites, the most common and investigated of them are alkaloids and saponins because of their biological properties.

**Figure 1:**
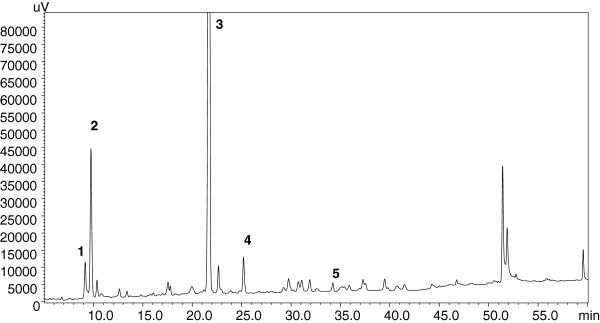
**HPLC-PDA analysis of *Nauclea latifolia *crude extract.** Peaks’ number are referred to those reported in Table 1.

Figure 1 reports the HPLC-PDA chromatogram of the crude extract from *N. latifolia*. Table 1 reports the list of the components tentatively identified by HPLC-PDA-ESI-qMS in the crude extract, together with their UV absorption maxima and MS data. Mass spectra, acquired in positive and negative ESI ionization (full scan) in general gave protonated [MH]^+^ or deprotonated [M–H]^-^ molecular ions respectively and, in positive mode, some molecular ion adducts [M + Na]^+^ and [M + K]^+^. The molecular mass of an unknown component was retained only when both [MH]^+^ and [M–H]^-^ ions were detected. The main components detected in the extracts belong to the class of indole alkaloids characteristic of *Nauclea* genus. In agreement with the literature data, strictosamide (3, RT = 21,675 min) seems to be the most abundant component in *N. latifolia* crude extract on the basis of its UV absorption maxima and the molecular ions in positive and negative ESI mode [27]. A less abundant component with UV maxima and molecular ions analogue to strictosamide that can be hypothesized as vincosamide (4, RT = 25,184 min) is also present. The presence of pumiloside (1, RT = 9,16 min) is confirmed by comparison of its UV maxima and molecular ions with those reported in the literature [28]. Compound 5 (RT = 34,224 min) has tentatively been identified as quinovic acid glycoside (a saponin) on the basis of its spectral data and those reported in literature [29]. Other components probably belonging to the same class (saponins or alkaloids) on the basis of their UV spectra were also detected; their identity is under investigation.

**Table 1:** ***Nauclea latifolia *crude extract composition obtained through HPLC-PDA-ESI-MS analysis**

**ID number**	**Compound name**	**(RT min)**	**UV max**	**Molecular weight**	**ESI +**	**ESI -**
1	Pumiloside	9.16	243	512	513	511
			315		535 [M + Na]^+^	
			328		551 [M + K ]^+^	
2	Alkaloid?	9.749	221	574	575	573
					597 [M + Na]^+^	
					613 [M + K ]^+^	
3	Strictosamide	21.675	223	498	499	497
					521 [M + Na]^+^	
					537 [M + K ]^+^	
					3337 [M + H-162]^+^	
4	Vincosamide	25.184	223	498	499	497
					521 [M + Na]^+^	
					537 [M + K ]^+^	
5	Quinovic acid glycoside?	34.224	--	632	633	631
						5587 [M-H-44]^-^

### *Nauclea latifolia* extract inhibits HSV-2 infectivity

**Figure 2:**
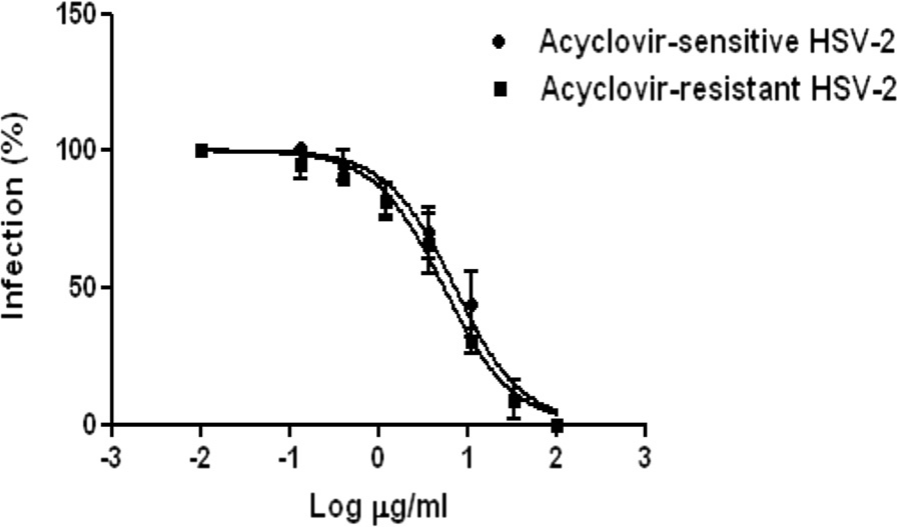
**Dose–response curve of *N. latifolia *extract against acyclovir-sensitive (●) and –resistant HSV-2 (■).** Vero cells were infected at a MOI of 0.001 pfu/cell, exposed for 24 h to different extract concentrations and tested in a plaque reduction assay. Data are presented as % of control. Values are mean ± SD of three separate determinations.

The antiviral activity of a *N. latifolia* extract was examined *in vitro* by plaque reduction assay against a clinical isolate of HSV-2 sensitive to acyclovir, and a mutant with phenotypic resistance to acyclovir. Vero cells were infected with virus in presence of different extract concentrations, ranging from 100 μg/ml to 0.13 μg/ml, and incubated for two hours at 37°C. The serial dilutions of plant extract were added again after the removal of the virus inoculum. The dose–response curves shown in Figure 2 demonstrate that the extract exerts a remarkable antiviral activity which is independent on the virus sensitivity to acyclovir. The IC_50_ values for the HSV-2 acyclovir-sensitive or resistant strain are 7.17 μg/ml (95% CI: 5.36 to 9.59) and 5.38 μg/ml (95% CI: 4.15 to 6.99) respectively. The antiviral activity of the extract was confirmed by a virus yield reduction assay, a more stringent test which allows multiple cycles of viral replication to occur before measuring the production of infectious viruses. Under this condition, the extract reduced the yield of the acyclovir-sensitive HSV-2 with an IC_50_ value of 1.46 μg/ml (95% CI: 1.07 to 1.91).

**Figure 3:**
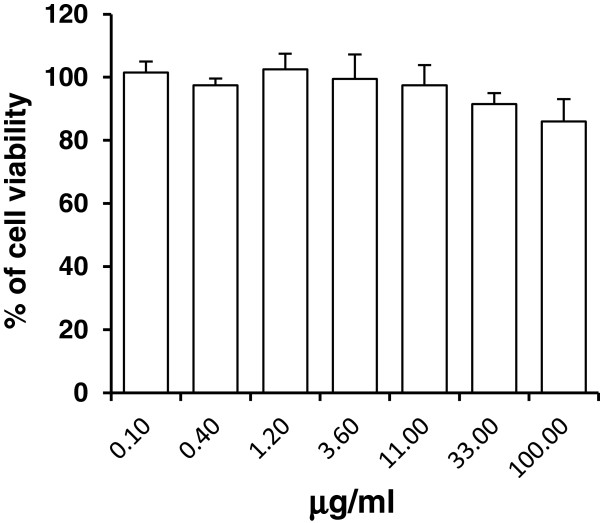
**Effect of *N. latifolia *extract on non-infected Vero cells viability, as a function of concentration at 48 h.** X axis: extract concentration, Y axis: cell viability (% of control). Each point represent the mean ± S.D. (n = 3).

To examine the effect on the cell viability, the extract was serially diluted and added to cell culture medium. For all cell culture experiments *N. latifolia* extract dilutions resulted in a DMSO concentration below 1% which had no effect on cells and viruses. After 48 hours of incubation at 37°C, viability of Vero cells was determined by MTS assay. As reported in Figure 3, the extract did not affect Vero cell viability at any concentration tested. The CC_50_ value was above 100 μg/ml, indicating that the antiviral activities observed were not due to cytotoxicity. Moreover, no changes in cell morphology compared with the control were observed by microscopic examination of the cell monolayers (data not shown). According to results of virus yield reduction assay, the selectivity index, a measure of the preferential antiviral activity of a drug in relation to its cytotoxicity, is above 68.4.

### Investigation of the major antiviral mechanism of action of extract

To explore if the extract exerts a direct virus-inactivating activity, a virucidal assay was performed with an extract concentration that reduces almost completely virus infection (IC_90_). To this aim, HSV-2 aliquots were incubated with 33 μg/ml of extract at 4°C or 37°C. After incubation, the samples were titrated on Vero cells at high dilutions at which the extract was not active. As reported in Table 2, this treatment did not produce a significant loss of HSV-2 infectivity, even at a concentration higher than its IC_50_. Therefore, since the antiviral activity was not exerted directly on HSV-2 particles before, we explored the effect of the extract on virus-cell interaction performing specific assays.

**Table 2:** **Virucidal assay: effect of preincubation of *N. latifolia *extract with HSV-2**

**Incubation condition**		**Extract**^**a**^	**Virus titer (PFU)**^**b**^
**Temp (°C)**	**Duration (h)**		
37	0	-	5.56 × 10^5^
37	0	+	3.52 × 10^5^
37	2	-	5.29 × 10^4^
37	2	+	3.42 × 10^4^
4	2	-	4.88 × 10^5^
4	2	+	6.03 × 10^5^

^a^Concentration: 33 μg/ml.

^b^The values are the means ± SD of data derived from three independent experiments performed in duplicate.

**Figure 4:**
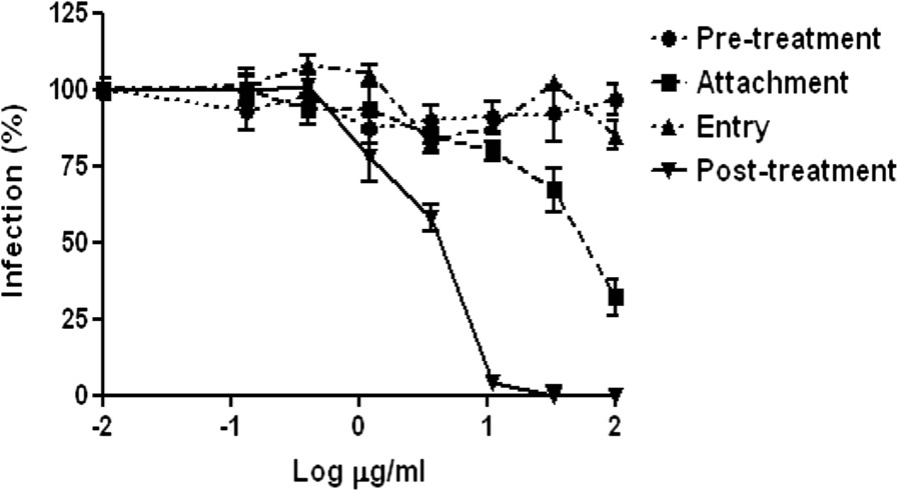
**Effect of *N. latifolia *treatment on plaque formation when added to cell cultures at different time intervals relative to virus infection.** Cells were pre-treated with extract prior to virus infection (pre-treatment), during the attachment period (attachment), during the entry period (entry) or after infection (post-treatment). Data are presented as % of control. Values are mean ± SD of three separate determinations.

To identify the stage of the virus replication cycle at which the extract acts, it was added to confluent Vero cells at different time intervals relative to virus infection. In all experiments cells infected with untreated virus were used as control and the inhibition of plaque formation by the extract was evaluated (Figure 4).

The pre-treatment assay shows that various doses of *N. latifolia* extract added 2 hours prior to virus infection and then being washed out did not exert inhibitory activity. Next, mixtures of HSV-2 and different concentrations of extract were added to Vero cells and incubated for two hours at 4°C to investigate the possible effect on the virus attachment to cells. A slight dose–response effect of extract on viral attachment was observed with an IC_50_ of 56.18 μg/ml (95% CI: 38.0 to 82.89). Since this value is more than two log higher than the IC_50_ value of heparin (0.14 μg/ml), a known inhibitor of HSV attachment (data not shown) [30], these data suggested that inhibition of attachment is not the main mechanism of action of *N. latifolia* extract. The entry assay was conducted to assess whether the extract prevents the viral penetration into the host cells. As shown in Figure 4 the extract does not act at this stage of the virus replication cycle. In contrast, when the extract was added to the overlay medium after entry of virus into Vero cells, a significant suppression of HSV-2 replication was observed with a IC_50_ value of of 3.63 μg/ml (95% CI: 2.6 to 5.08). The strong inhibitory effect of the tested extract in post-treatment assay was also confirmed by the virus yield reduction assay with an IC_50_ of 8.23 μg/ml (95% CI: 5.33 to 12.70) (Figure 4).

## Discussion

*N. latifolia* belongs to Rubiaceae*,* a large family comprising more than 630 genera and about 13000 species of plants. Some of them, including *N. latifolia,* are used in sub-Saharan traditional medicine to treat several diseases suggesting that they may represent a natural source of pharmacologically active substances [31]*.* Although a number of studies indicated a potential therapeutic use of *N. latifolia*[16-18,32-34] at the best of our knowledge its antiviral activity has not been investigated so far. To explore the antiviral potential of *N. latifolia,* HSV-2 was chosen as a viral model because this virus has a strong impact on HIV transmission and acquisition [9,35,36]. Indeed, it has been proposed that in areas with a high HIV and HSV-2 prevalence, such as sub-Saharan Africa, antiviral therapy against HSV-2 could have a population-level impact on the global HIV epidemic [8,9,37,38]. Within a study to screen the biological activity of plants native of Central Africa, the present study reports for the first time a remarkable anti-HSV-2 activity of a CH_2_Cl_2_-MeOH root barks extract of *N. latifolia*. Although a crude extract may contain several molecules endowed with antiviral activity each acting through a different mechanism some preliminary conclusions can be drawn on the main mode of antiviral action. Further in depth study on the *N. latifolia* extract composition are under way. The virucidal assay rules out the possibility that the antiviral activity of the extract is exerted by a direct inactivation of the virus particle. Time of addition experiments revealed that the strongest inhibition occurs when the extract is added post-infection. These findings indicate that the main mechanism of action is not the inhibition of virus attachment or entry. Moreover, the acyclovir-resistant HSV-2 strain was as susceptible to *N. latifolia* extract as the acyclovir-sensitive strain, suggesting that the mode of antiviral action is different from that of acyclovir. This latter feature, along with a low cytotoxicity and a favourable selectivity index make the *N. latifolia* extract a promising starting material for a bioguided-fractionation aimed at identifying anti-HSV-2 compounds with a novel mechanism of action that can be used against acyclovir-resistant HSV-2 strains.

## Conclusion

The results of the present study are in line with the use of *N. latifolia* for treatment of viral diseases in African folk medicine. IC_50_ values allow us to believe that *N. latifolia* roots constitute a natural source of anti-HSV2 substances, although further work remains to be done in order to isolate the active principle and elucidate its mechanism of action, or to develop a phytodrug from *N. latifolia* CH2Cl2/MeOH extract.

## Abbreviations

HSV: Herpes simplex virus; CH2Cl2: Methylene chloride; MeOH: Methanol; HIV: Humane immune-deficiency virus; IC50: Inhibitory concentration producing 50% reduction in plaque formation; HPLC: High pressure liquid chromatography; PDA: Phtodiode array; MS: Mass spectroscopy; APCI: Atmospheric pressure chemical ionization; ESI: Electrospray ionization; CDL: Desolvation line; MEM: Eagle’s minimal essential medium; MTS: 3-(4,5-dimethylthiazol-2-yl)-5-(3-carboxymethoxyphenyl)-2-(4-sulfophenyl)-2H-tetrazolium; MOI: Multiplicity of infection; DMSO: Dimethyl sulfoxide; PFU: Plaque forming unit; SI: Selectivity index; CI: Confidence interval.

## Competing interests

The authors declare that they have no competing interests.

## Authors’ contributions

MD performed the antiviral assays, produced the acyclovir resistant HSV-2 strain, did the statistical analysis of the data and drafted the manuscript. HMN collected ethnobotanical data on the plant as well as the plant itself in the field, prepared the crude extract, participated in antiviral assays and manuscript drafting. RANN, DG and FFB were involved in conception and supervision (ethnobotanical data and plant collection in the field) of this work, and deep manuscript revision. ATT provided laboratory means for preparation of the crude extract and revised the manuscript. RR and VC did the virus time of addition assays. PR, CC and CB performed the phytochemical analyses on *N.latifolia* crude extract and interpreted the obtained data. DL supervised the work on the whole and provided laboratory means for the antiviral assays. All authors contributed substantially to the present work then read and approved the final manuscript.

## Authors’ information

MD is an assistant Professor working in the Laboratory of Molecular Virology, University of Turin, Italy.

HMN is a PhD student working in Laboratory of Microbiology and Antimicrobial Substances, University of Dschang, Cameroon. She was awarded a funding by AIRES-Sud program for an internship in the Laboratory of Molecular Virology at the University of Turin to learn the techniques of antiviral assays.

RANN is Associate Professor, Head of Department of Biochemistry at the University of Douala, Cameroon; she coordinated the AIRES-Sud funded project number 7082. She is also one of miss Magnifouet’s PhD supervisors.

DG is Associate Professor, Senior lecturer at the Department of Biochemistry, University Dschang, Cameroon; he is moreover one of miss Magnifouet’s PhD supervisors.

ATT is Senoir researcher at the Laboratory of Phytochemistry of the Institute of Medical Research and Medicinal Plants Studies in Yaoundé, Cameroon.

VC is PhD student working in the Laboratory of Molecular Virology, University of Turin, Italy.

RR is Master of Science; she carried out her research in the Laboratory of Molecular Virology, University of Turin, Italy.

CC is an assistant Professor working in the laboratory of Phytoanalysis at the Department of Drug Science and Technology, University of Turin.

FFB is Associate Professor, Head of the Laboratory of Phytobiochemistry and medicinal plants study, University of Yaoundé, Cameroon; besides he is one of miss Magnifouet’s PhD supervisors.

PR is Professor, Senior lecturer working in the Laboratory of Phytoanalysis at the Department of Drug Science and Technology, University of Turin, Italy.

CB is Professor, Head of the Laboratory of Phytoanalysis, at the Department of Drug Science and Technology,University of Turin, Italy. He is Cagliero’s research supervisor.

DL is Professor, Head of the Laboratory of Molecular Virology, University of Turin, Italy. He is also Donalisio’s Cagno’s and Rovito’ research supervisor.

## Pre-publication history

The pre-publication history for this paper can be accessed here:

http://www.biomedcentral.com/1472-6882/13/266/prepub
